# Effect of 14-Week Supplementation of Highly Purified Policosanol (Raydel^®^) and a Sugar Cane Extract Powder (SCEP) on Dyslipidemia and Oxidative Variables in Hyperlipidemic Zebrafish: Insight into Liver, Kidney, and Brain Health

**DOI:** 10.3390/cimb47050354

**Published:** 2025-05-13

**Authors:** Kyung-Hyun Cho, Ashutosh Bahuguna, Sang Hyuk Lee, Ji-Eun Kim, Yunki Lee, Cheolmin Jeon

**Affiliations:** Raydel Research Institute, Medical Innovation Complex, Daegu 41061, Republic of Korea

**Keywords:** dyslipidemia, fatty liver, glucose, 4-hydroxynonenal, interleukin 6, policosanol, sugarcane-extract powder, senescence, zebrafish

## Abstract

The efficacy of Cuban sugarcane-extracted policosanol (Raydel^®^), a purified blend of eight long-chain aliphatic alcohols, was compared to copycat sugarcane-extract powder (SCEP) to assess their effects on dyslipidemia, oxidative stress, and vital organs of zebrafish under the influence of a high-cholesterol diet (HCD). Zebrafish were fed with HCD (final 4%, *w*/*w*) or HCD infused with policosanol (PCO, final 1%, *w*/*w*) or SCEP (final 1%, *w*/*w*). Post 14-week consumption, blood and organs were harvested and processed for various biochemical, histological, and immunohistochemical (IHC) examinations, and fluorescent staining. Following 14-week consumption, the PCO-supplemented group exhibited higher zebrafish survival probability than the SCEP-supplemented group. Both PCO and SCEP substantially impacted the HCD-disrupted plasma lipid profile; however, PCO supplementation exhibited a significantly better effect than SCEP. Similarly, PCO supplementation significantly improved the blood glucose level, hepatic function biomarkers, and oxidative-antioxidant balance disturbed by HCD. PCO supplementation displayed a substantial inhibitory effect against HCD-induced fatty liver changes, nephromegaly, and cellular senescence. Likewise, PCO effectively protected the brain against HCD-induced apoptosis and accumulation of 4-hydroxynonenal (4-HNE); in contrast, SCEP supplementation showed almost no effect in reducing such adverse changes. The comparative findings between PCO and SCEP highlight the protective effects of PCO against HCD-induced oxidative stress and dyslipidemia via the enhancement of antioxidant markers, leading to protection of the liver, kidney, and brain, while SCEP failed to achieve similar outcomes.

## 1. Introduction

Dyslipidemia is a metabolic disorder described as abnormally high total cholesterol (TC), triglycerides (TG), low-density lipoprotein cholesterol (LDL-C), and diminished high-density lipoprotein cholesterol (HDL-C) levels [1,2,3]. Over the past few decades, the presence of dyslipidemia has increased across the globe [1], and it has been estimated by the World Health Organization (WHO) that nearly 40% of the global population has an elevated blood cholesterol concentration [4]. A passive lifestyle, obesity, altered food habits, and genetic factors are among the common causes of dyslipidemia [1,5]. Notably, 50–70% of individuals with obesity have dyslipidemia, highlighting a strong connection between the two conditions [6]. Dyslipidemia is a major factor in the pathogenesis of numerous diseases, particularly exerting a strong influence on cardiovascular disease (CVD) progression [1,7]. Beyond its role in cardiovascular disease, dyslipidemia promotes the progression of renal failure in individuals with kidney disease [8]. Also, dyslipidemia contributes to insulin resistance mediated by various mechanisms, including a detrimental effect on pancreatic β-cells, resulting in hyperglycemia [9]. Furthermore, dyslipidemia and oxidative stress are strongly associated with each other [10,11] and lead to several adverse events.

For the curative treatment of dyslipidemia, statins are the first line of drug treatment; however, adverse effects such as weakness, depression, and muscular pain are commonly associated with the consumption of statins [4,12,13]. In contrast to statins, many rigorous clinical studies have documented herbal formulations as safe and effective lipid-lowering agents [4]. Also, the International Lipid Expert Panel suggested herbal monomers and their derivatives as safe and tolerable agents [14] and recommended their use as a substitute for statins, particularly in the statin-intolerant population [15].

A range of herbal and nutraceuticals, such as phytoestrogens, policosanol, phytosterols, and red yeast rice extract, demonstrated substantial potential in managing dyslipidemia [16]. Among the varied nutraceuticals, policosanol has gained substantial attention as a lipid-lowering agent, including its effect on blood pressure and as an anti-platelet aggregation agent [17]. In addition to its effect on dyslipidemia, policosanol has been reported to effectively lower blood glucose levels [17,18], reduce the generation of reactive oxygen species (ROS) [19], and decrease lipid peroxidation markers, such as malondialdehyde (MDA) [20]. Policosanol is a blend of long-chain aliphatic alcohols (LCAA) extracted from various sources, including rice bran, wheat germ, insects, and sugarcane [17,21]. However, the composition of policosanol varies greatly based on the source material, its geographical location, and the method of extraction, greatly impacting the functionality of policosanol [17,22]. The accumulated literature has documented the functional variation of policosanol with respect to dyslipidemia [17]. Nonetheless, most studies suggest an effective role of policosanol in countering dyslipidemia [17]. The first authentic policosanol moiety was extracted from Cuban sugarcane (*Saccharum officinarum* L.) wax in the early 1990s and characterized as a blend of eight LCAA of C24 (0.1–2.0%), C26 (3.0–10.0%), C27 (0.1–3.0%), C28 (60–70%), C29 (0.1–2.0%), C30 (10–15%), C32 (5–10%), and C34 (0.1–5.0%) [23]. Subsequently, different policosanols were extracted from different origins and source materials and using different methods. Currently, over 40 policosanol-based products to mimic functional food are available globally, yet their efficacy varies significantly and is not comprehensively established [19]. Moreover, the availability of certain copycat products with unverified composition and lacking safety data has undermined the credibility of authentic policosanol formulations. In Korea, exclusively Cuban sugarcane wax-derived policosanol was approved by the Ministry of Food and Drug Safety (MFDS) [formerly the Korea Food and Drug Administration (KFDA)] as a health-functional food [24].

Very recently, in 2024, almost a dozen sugarcane extract powders (SCEPs) have appeared in the Korean market, as sugar-processed products, under general food categories, such as candy, sugar syrup, chewing gum, and oligosaccharides. Since these products are classified as general food, they are not bound to disclose the exact composition of their ingredients or require approval from MFDS, unlike functional foods such as policosanol (Raydel^®^), which are strictly regulated in terms of origin and active ingredient composition. Despite being registered under general food categories, many such processed sugar food products claim to mimic registered functional food. For instance, one type of SCEP, Dr. Lean (Cosnature, Seoul, Republic of Korea), purporting to be a functional food, was shown to contain sugarcane wax alcohol amalgamated with red yeast rice (RYR) [25]. However, these claims lack scientific validation, making it essential to assess their efficacy through rigorous research.

Given the above, the present study aimed to compare the effect of 14 weeks consumption of Cuban sugarcane wax-purified policosanol (Raydel^®^) and a SCEP (Dr. Lean) on high-cholesterol-diet-induced dyslipidemia in the zebrafish. Additionally, this study assessed the comparative impact of the policosanol and SCEP (Dr. Lean) on blood glucose levels, oxidative stress, antioxidant status, liver function, fatty liver changes, inflammation, and histological alterations in the kidney and brain of the hyperlipidemic zebrafish.

## 2. Materials and Methods

### 2.1. Materials

Raydel^®^ policosanol (PCO), a typical mixture of eight long-chain aliphatic alcohols (LCAA, C24-C34) purified from sugarcane wax originating from Cuba, was provided on a complementary basis by Raydel^®^ Australia Pty, Ltd., (Thornleigh, NSW, Australia). Sugarcane extract powder (SCEP, Dr. Lean) containing ground whole sugarcane amalgamated with red yeast rice (RYR) and other constituents was purchased from Cosnature (Seoul, Republic of Korea). A detailed specification of the policosanol (batch: 310030324) and SCEP (batch: 2400023) is provided in Supplementary Materials Tables S1 and S2. Primary antibodies against interleukin 6 (IL-6, ab9324) and 4-hydroxynonenal (4-HNE, ab45506) were purchased from Abcam (Cambridge, UK), respectively. The specification of the other chemicals used is provided in Supplementary Materials Section S1.

### 2.2. Zebrafish Husbandry

The 16-week-old adult zebrafish were maintained in a water tank equipped with a recycling water system following the standard Animal Use and Care guidelines adopted by the Raydel Research Institute (date of approval 27 July 2023, approval code RRI-23-007). During the aquaculture, the zebrafish were maintained on an alternative 14 h light and 10 h dark cycle and fed with a normal tetrabit diet (ND, Tetrabit Gmbh D49304, Melle, Germany).

### 2.3. Preparation of Different Dietary Formulations

The ND was used as source material to formulate the different diets. For the preparation of a high-cholesterol diet (HCD), ND was blended with 4% (*w*/*w*) cholesterol. The HCD was mixed with a crushed tablet of policosanol (Raydel^®^, final 1%, *w*/*w*) or sugarcane extract powder (Dr. Lean, final 1%, *w*/*w*) to prepare the HCD-supplemented diets, which were named HCD + PCO and HCD + SCEP, respectively. The rationale behind the selection of the used amount was based on a previous study [26], which demonstrated 1.0% PCO provided the most favorable results. To maintain consistency, an equal amount (1.0%) of SCEP was used for comparative analysis.

### 2.4. Supplementation of Different Dietary Formulations

**Figure 1:**
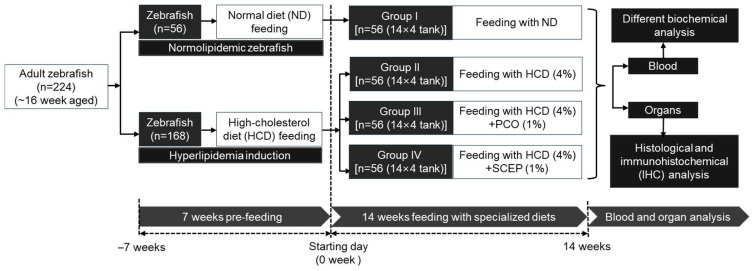
Study layout of zebrafish feeding with different diets. Abbreviations: ND: normal tetrabit-fed group; HCD: high-cholesterol diet group; HCD + PCO or SCEP: high-cholesterol diet infused with policosanol (Raydel^®^) or sugarcane extract powder (Dr. Lean) group.

Adult zebrafish (*n* = 224) were randomly allocated into four groups (*n* = 56/group), named ND (group I), HCD (group II), HCD + PCO (group III), and HCD + SCEP (group IV). Zebrafish in group I were fed the ND diet, while those in groups II, III, and IV were fed HCD, HCD + PCO, and HCD + SCEP diets, respectively. Zebrafish (*n* = 56) of each group were distributed into four separate tanks (*n* = 14/tank) and supplemented with the specified diets for 14 weeks. Zebrafish in each tank (*n* = 14) received 140 mg of the respective diets, administered twice daily [morning (9 a.m.) and evening (6 p.m.)], cumulating to 280 mg/tank (equivalent to 20 mg/day/zebrafish). The percentage of food consumption among all the groups was measured using a previously described method [27], using the formula: [(cumulative amount of food given per +group (mg) − remaining amount of food (mg))/cumulative amount of food given (mg)] × 100. Across all the groups, nearly 90–100% feed consumption was observed within 30 min of feed supply, suggesting that the zebrafish had no preferences towards the different dietary preparations. Notably, zebrafish in groups (II to IV) were maintained only on the HCD diet for 7 weeks (before initiating the experimentation) to induce hyperlipidemia, following a methodology resembling an earlier described method [19,27]. Figure 1 depicts the experimental arrangements.

During the 14 weeks, zebrafish survivability in each group was assessed and recorded. The weight of the zebrafish was assessed on the starting day (day 0) and finally recorded at the end of 14-week supplementation in the respective groups.

### 2.5. Collection of Blood and Organs

Post 14 weeks of supplementation with each designated diet, zebrafish from the different groups were euthanized, and blood was immediately collected. Zebrafish across the groups were deprived of food overnight (~14 h) before the collection of blood. Importantly, samples were collected from the zebrafish across the groups on the same day between ~9 a.m. and 10.30 a.m. to minimize circadian and feeding-related variability. For the blood collection, 14 zebrafish/tanks of a specified group were sacrificed, and ~2–5 μL blood from each zebrafish was collected and pooled. The pooled blood from 14 zebrafish/tank (for the specified group) was mixed with phosphate-buffered saline (PBS)-ethylenediaminetetraacetic acid (EDTA, final concentration, 1 mM) in 2:3 (*v*/*v*) ratio and centrifuged to obtain the plasma.

The different organs (liver, kidney, and brain) from the sacrificed zebrafish were surgically removed and preserved in 10% formalin for further use.

### 2.6. Analysis of Blood Lipid Profile, Hepatic Function Biomarkers, Glucose, and Antioxidant Variables

Total cholesterol (TC), triglycerides (TG), high-density lipoprotein cholesterol (HDL-C), and the blood hepatic function biomarkers aspartate aminotransferase (AST) and alanine aminotransferase (ALT) levels among the different groups were quantified using commercial assay kits following the manufacturers’ instructions. A detailed procedure is provided as Supplementary Materials Section S2.

The blood glucose level was quantified by a digital glucose meter (AccuCheck, Roche, Basel, Switzerland). The plasma malondialdehyde (MDA) level was quantified by a colorimetric method [28] by blending plasma (20 μL, equivalent to 1 mg/mL protein) with trichloroacetic acid (50 μL) and thiobarbituric acid (100 μL). The absorbance at 560 nm was recorded after 10 min incubation at 95 °C.

The ferric ion reduction ability (FRA) was assessed by mixing the plasma (20 μL, equivalent to 1 mg/mL protein) with an FRA reagent (180 μL) [28]. After 60 min incubation, absorbance at 593 nm was recorded. For the detection of paraoxonase (PON) activity [28], plasma (40 μL, equivalent to 1 mg/mL protein) was mixed with 150 mg/mL paraoxon ethyl (160 μL) following 2 h incubation; absorbance was noted at 415 nm. The enzyme activity was expressed as μU/L/min using the formed product (*p*-nitrophenol) molar absorbance coefficient (ε = 1.7 × 10^3^/M/cm).

For quantification of the sulfhydryl content, the plasma (50 μL, equivalent to 1 mg/mL protein) was mixed with 0.4% of 5,5-dithio-bis-(2-nitrobenzoic acid) (50 μL). After 2 h incubation at room temperature, absorbance at 412 nm was recorded, and the results are expressed as nmol/mg of protein quantified using the formed product (5-thiol-2-nitrobenzoic acid) extinction coefficient (ε = 1.36 × 10^4^/M/cm).

For all the biochemical analyses, four replicates (*n* = 4)/group were used and the mean value ± SEM of the four replicates was used to depict the results

### 2.7. Histological Analysis

For the histological analysis, tissue from the liver, kidney, and brain was implanted into FSC22 frozen material (Leica, Nussloch, Germany) and processed for sectioning using a cryo-microtome (Leica CM-1510S, Nussloch, Germany). An individual tissue section (7 μm thick) obtained from the liver, kidney, and brain was processed for histological analysis using hematoxylin and eosin (H&E) staining following a previously described method [29].

For the fatty liver changes, the hepatic section (7 μm thick) was covered with 0.3% Oil Red O solution and incubated at 60 °C for 5 min. The stained section was washed with 60% isopropanol and analyzed under the microscope.

### 2.8. Cellular Senescence, Dihydroethidium (DHE), and Acridine Orange (AO) Fluorescent Staining

Senescence-associated β-galactosidase staining was performed to detect senescence in the liver, kidney, and brain [30]. The tissue section (7 μm thick) was covered with 0.1% 5-bromo-4-choloro-3-indolyl-β-D-galactopyranoside solution and incubated in a moist environment for 16 h following microscopic examination to detect the blue-stained senescence-positive cells.

Reactive oxygen species (ROS) in the tissue section were detected by dihydroethidium (DHE) fluorescent staining [31]. Briefly, the tissue section (7 μm thick) was covered with 250 μL of DHE (30 μm). After 5 min incubation, the tissue section was washed and analyzed under a fluorescent microscope at the excitation and emission wavelengths of 585/615 nm. Apoptosis in the brain tissue section was detected by acridine orange (AO) fluorescent staining [31]. Briefly, the brain tissue section (7 μm thick) was covered with 250 μL AO (5 μg/mL) for five minutes. Afterward, the tissue section was washed and analyzed under a fluorescent microscope at the excitation and emission wavelengths 505/535 nm to detect AO fluorescent intensity.

### 2.9. Immunohistochemical (IHC) Staining

Interleukin 6 (IL-6) was detected in the hepatic tissue by IHC staining [32] by covering the hepatic section with 200× diluted IL-6-specific primary antibody (Abcam ab9324). Following 16 h incubation at 4 °C, the section was developed with the EnVision + system HRP polymer kit (code K4001, Dako, Glostrup, Denmark) and visualized under a microscope.

For the detection of 4-hydroxynonenal (4-HNE), the brain section was flooded with 200× diluted anti-4HNE antibody (Abcam, ab48506). After 16 h incubation at 4 °C, the section was treated with Alexa Fluor^TM^ (fluorescently tagged) secondary antibody (AB_2534073, Invitrogen, Waltham, MA, USA) specific against the anti-4HNE antibody. Finally, the section was visualized under a fluorescent microscope.

### 2.10. Statistical Analysis

The statistical differences in the multiple groups were established by conducting a one-way analysis of variance (ANOVA) following Tukey’s post hoc analysis using version 29, Statistical Package for the Social Science (SPSS software package, Chicago, IL, USA). Prior to performing the ANOVA, the data distribution normality was examined using the Kolmogorov–Smirnov test.

## 3. Results

### 3.1. Zebrafish Survivability and Body Weight

**Figure 2:**
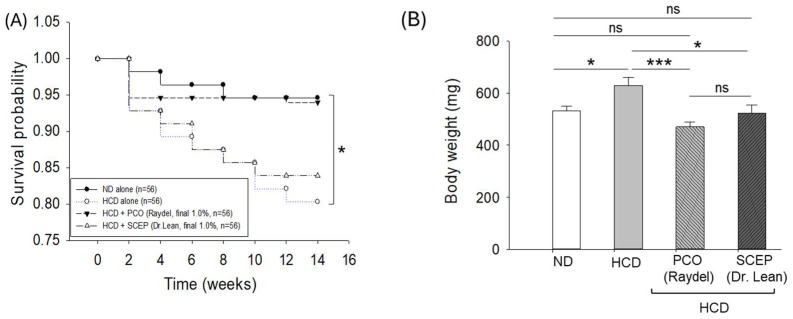
Effect of high-cholesterol diet (HCD) infused with policosanol (PCO) or sugarcane extract powder (SCEP) on the survivability and body weight of zebrafish. (**A**) Kaplan–Meier survival probability curve; * highlights statistical difference (*p* < 0.05) at log-rank: χ^2^ = 8.56. (**B**) Body weight of the adult zebrafish at week 0 and 14-weeks consumption of the respective diets. The sign * (*p* < 0.05), *** (*p* < 0.001) indicates statistical difference in body weight changes compared to the HCD group. “ns” represents a non-significant difference between the groups. Abbreviations: ND: normal tetrabit-fed group; HCD: high-cholesterol diet group; HCD + PCO or SCEP: high-cholesterol diet infused with policosanol (Raydel^®^) or sugarcane extract powder (Dr. Lean) group.

The survival probability outcomes based on Kaplan–Meier survival analysis revealed a significant (log-rank: χ^2^ = 8.56, *p* = 0.036) survival difference among the groups. The survivability of zebrafish was hampered by the consumption of HCD, as reflected by the least survival probability (0.8) after 14 weeks of consumption, while the ND group showed the highest survival probability (0.95) (Figure 2A). The consumption of PCO effectively prevented HCD-induced zebrafish death displayed by a survival probability (0.95) similar to the ND group. Contrary to PCO, SCEP was associated with a poor zebrafish survival probability (0.84) against HCD-induced zebrafish death after 14 weeks of supplementation.

The repeated measures ANOVA employing multivariate tests (e.g., Pillai’s Trace, Wilks Lambda, Hotelling’s Trace, and Roy’s Largest Root) revealed a significant effect of time (F = 435.85, *p* < 0.001) and an interaction of time with different dietary formulations (F = 4.86, *p* = 0.004) on body weight changes from week 0 to week 14 (Supplementary Materials Figure S1). Also, the different diets had a significant effect (F = 4.57, *p* = 0.005) on the body changes; however, the most notable changes were observed in the HCD group, which showed a 1.9-fold elevation in body weight. In contrast, the PCO-supplemented group displayed an at least 1.5-fold enhanced body weight compared to the body weight of week 0 (Supplementary Materials Figure S1). The multiple comparison analysis of the body weight at week 14 showed a significant 25.2% (*p* < 0.001) reduced body weight in the PCO-supplemented group compared to the HCD group (Figure 2B). Similarly, the ND and SCEP-supplemented groups exhibited ~16% (*p* < 0.05) lower body weight than the HCD group. However, a non-significant (*p* > 0.05) difference in body weight was observed between the ND, PCO, and SCEP-supplemented groups at week 14 (Figure 2B).

### 3.2. Blood Lipid Profile, Hepatic Function Biomarkers, and Glucose Level

**Figure 3:**
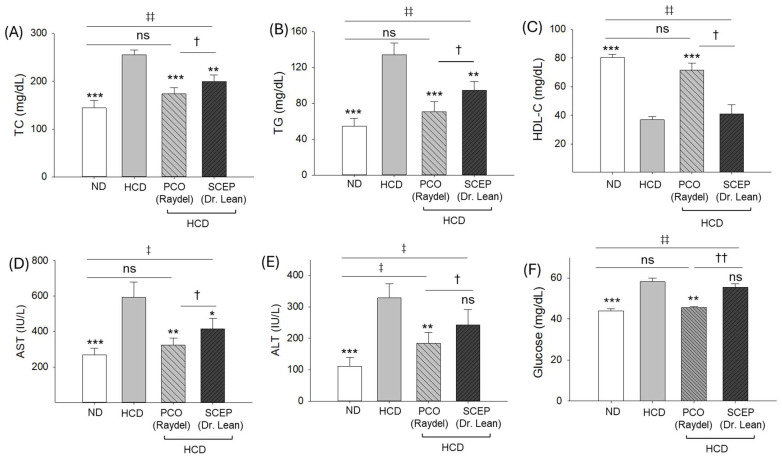
Blood lipid profile, hepatic function biomarkers, and glucose level of zebrafish fed with a high-cholesterol diet (HCD) infused with policosanol (PCO) or sugarcane extract powder (SCEP) during 14 weeks of consumption. (**A**) Total cholesterol (TC), (**B**) triglycerides (TG), (**C**) high-density lipoprotein cholesterol (HDL-C), (**D**) aspartate aminotransferase (AST), (**E**) alanine aminotransferase (ALT), and (**F**) blood glucose level. Abbreviations: ND: normal tetrabit-fed group; HCD: high-cholesterol diet group; HCD + PCO or SCEP: high-cholesterol diet infused with policosanol (Raydel^®^) or sugarcane extract powder (Dr. Lean) group. The signs * (*p* < 0.05), ** (*p* < 0.01), *** (*p* < 0.001) indicate statistical difference vs. HCD group; ^‡^ (*p* < 0.05), ^‡‡^ (*p* < 0.01) indicate statistical difference vs. ND group, and ^†^ (*p* < 0.05), ^††^ (*p* < 0.01) indicate statistical difference vs. PCO group. “ns” represents a non-significant difference between the groups.

The blood lipid profile showed elevated levels of TC (255.2 ± 10.1 mg/dL) and TG (134.3 ± 13.2 mg/dL) and reduced HDL-C (36.9 ± 2.2 mg/dL) level in the HCD group (Figure 3A–C). The consumption of PCO and SCEP substantially reduced HCD-elevated TC and TG; however, PCO exhibited a significantly (*p* < 0.05) better effect compared to SCEP. A 173.7 ± 12.8 mg/dL TC and 70.8 ± 11.1 mg/dL TG level was observed in the PCO-supplemented group, which was significant at 13.1% and 25.3% lower than the TC (199.8 ± 13.5 mg/dL) and TG (94.8 ± 9.7 mg/dL) levels observed in the SCEP-supplemented group. The HCD-diminished HDL-C (36.9 ± 2.2 mg/dL) level was significantly elevated by the supplementation of PCO (71.7 ± 4.8 mg/dL), while consumption of SCEP (41.0 ± 6.4 mg/dL) did not enhance the HDL-C level significantly. Notably, the effect of PCO on the TC, TG, and HDL-C levels was statistically similar, with the ND group demonstrating PCO efficacy in reverting HCD-induced dyslipidemia to basal levels.

The highest AST (593.4 ± 86.4 mg/dL) and ALT (328.9 ± 44.3 mg/dL) levels were detected in the HCD group, which was significantly (*p* < 0.01) decreased to 324.1 ± 39.9 mg/dL and 183.8 ± 34.8 mg/dL, respectively, following PCO supplementation (Figure 3D,E). Unlike PCO, no significant effect of SCEP in reducing the HCD-elevated ALT level was observed; however, a significantly (*p* < 0.05) 1.4-fold lower AST level (416.2 ± 59.4 mg/dL) was observed in response to SCEP compared to the HCD group, though the effect was ~28% (*p* < 0.05) inferior compared to the AST level observed in the PCO-supplemented group.

A maximum blood glucose level (58.2 ± 1.6 mg/dL) was observed in the HCD-supplemented group that was ~32% (*p* < 0.001) more than the basal glucose level detected in the ND control group (44.1 ± 0.9 mg/dL) (Figure 3F). Supplementation of PCO reduced the HCD-elevated blood glucose level (45.5 ± 0.6 mg/dL) and was equivalent to the glucose level observed in the ND-control group. A non-significant effect (*p* > 0.05) of SCEP supplementation in reducing the HCD-elevated blood glucose level was observed.

### 3.3. Blood Oxidative and Antioxidant Variables

**Figure 4:**
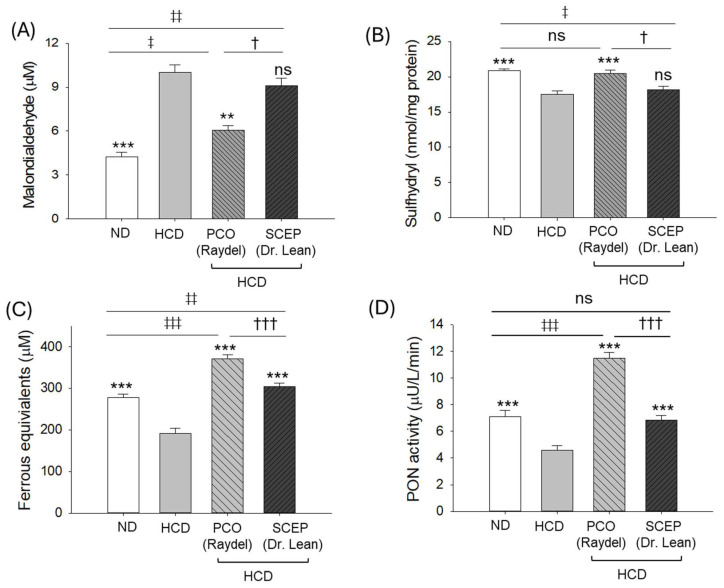
Blood oxidative and antioxidant variables of zebrafish fed with a high-cholesterol diet (HCD) infused with policosanol (PCO) or sugarcane extract powder (SCEP) during 14 weeks of consumption. (**A**) Malondialdehyde level (MDA), (**B**) sulfhydryl content, (**C**) ferric ion reduction assay (FRA), (**D**) paraoxonase (PON) activity. Abbreviations: ND: normal tetrabit-fed group; HCD: high-cholesterol diet group; HCD + PCO or SCEP: high-cholesterol diet infused with policosanol (Raydel^®^) or sugarcane extract powder (Dr. Lean) group. The signs ** (*p* < 0.01), *** (*p* < 0.001) indicate the statistical difference vs. HCD group; ^‡^ (*p* < 0.05), ^‡‡^ (*p* < 0.01), and ^‡‡‡^ (*p* < 0.001) indicate statistical difference vs. ND group; and ^†^ (*p* < 0.05), ^†††^ (*p* < 0.001) indicate statistical difference vs. PCO group. “ns” represents a non-significant difference between the groups.

A significantly 2.4-fold greater elevated plasma malondialdehyde (MDA) level was observed in the HCD-supplemented group (10.1 ± 0.5 μM) compared to the MDA level observed in the ND group (4.2 ± 0.3 μM). The supplementation of PCO substantially reduced the HCD-elevated MDA level by 1.6-fold (Figure 4A). A non-significant effect of SCEP was observed in curtailing the HCD-triggered MDA level.

A significantly diminished plasma sulfhydryl content was quantified in the HCD-supplemented group (17.5 ± 0.5 nmol/mg protein) compared to the ND group (20.9 ± 0.3 nmol/mg protein) (Figure 4B). The supplementation of PCO showed a 16.5% elevated (20.5 ± 0.5 nmol/mg protein, *p* < 0.001) sulfhydryl content compared to the HCD-supplemented group. A non-significant effect of SCEP was observed in elevating the HCD-impaired plasma sulfhydryl content.

Compared to the ND group, ~1.5-fold reduced ferric ion reduction activity (FRA) was observed in the HCD consumption group, which was significantly improved by 1.9-fold (*p* < 0.001) and 1.6-fold (*p* < 0.001) following PCO and SCEP supplementation, respectively (Figure 4C). However, compared to SCEP, 1.2-fold (*p* < 0.001) higher FRA was observed in response to PCO, confirming the superiority of PCO over SCEP in improving HCD-diminished plasma FRA activity.

Similarly, HCD adversely affected PON activity, which was ~1.6-fold (*p* < 0.001) lower than the basal paraoxonase (PON) activity observed in the ND group (Figure 4D). Both PCO and SCEP supplementation effectively prevented the HCD-diminished PON activity, reflected in 2.5-fold and 1.5-fold higher PON activity in the PCO- and SCEP-supplemented groups compared to the HCD group. Interestingly, PCO had the most influential impact on PON activity, which was ~1.6-fold (*p* < 0.001) higher than the PON activity observed in the ND- and SCEP-supplemented groups.

### 3.4. Morphology and Organ/Body Weight Analysis

**Figure 5:**
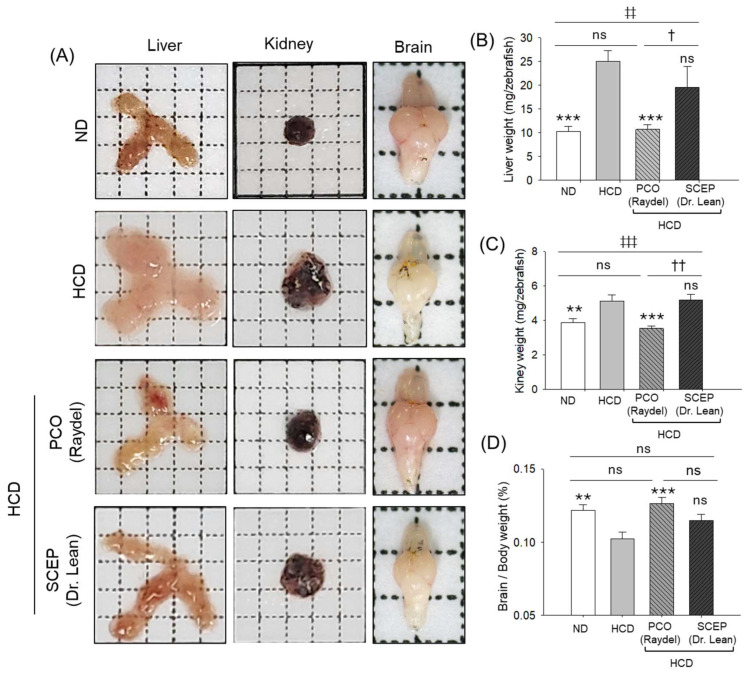
Different organ (liver, kidney, and brain) morphology and weight obtained from zebrafish fed with a high-cholesterol diet (HCD) infused with policosanol (PCO) or sugarcane extract powder (SCEP) during 14 weeks of consumption. (**A**) Representative images of the liver, kidney, and brain. (**B**–**D**) Liver, kidney, and brain weight, respectively. Abbreviations: ND: normal tetrabit-fed group; HCD: high-cholesterol diet group; HCD + PCO or SCEP: high-cholesterol diet infused with policosanol (Raydel^®^) or sugarcane extract powder (Dr. Lean) group. The signs ** (*p* < 0.01), *** (*p* < 0.001) indicate statistical difference vs. HCD group; ^‡‡^ (*p* < 0.01), ^‡‡‡^ (*p* < 0.001) indicate statistical difference vs. ND group, and ^†^ (*p* < 0.05), ^††^ (*p* < 0.01) indicate statistical difference vs. PCO group. “ns” represents a non-significant difference between the groups.

The morphological analysis of the liver obtained from the HCD group showed enlargement in size with a notable ~2.4-fold (*p* < 0.001) higher liver/body weight compared to the ND group (Figure 5A,B). HCD-induced hepatomegaly and elevated liver/body weight were prevented by supplementation of PCO with a ~2.3-fold (*p* < 0.001) reduced liver/body weight, which was analogous to the ND group. Similarly, HCD adversely impacted kidney morphology and weight, which was prevented by supplementation of PCO, reflected in 1.4-fold (*p* < 0.001) reduced kidney/body weight compared to the HCD-consumed group (Figure 5A,C). The brain morphology did not show much difference across the groups; however, the brain/body weight was ~1.2-fold (*p* < 0.001) diminished in the HCD-supplemented group compared to the ND and PCO groups (Figure 5A,D). Surprisingly, SCEP had a non-significant effect on HCD-provoked hepatomegaly, nephromegaly, and organ/body weight.

### 3.5. Liver Histology and Immunohistochemistry (IHC)

**Figure 6:**
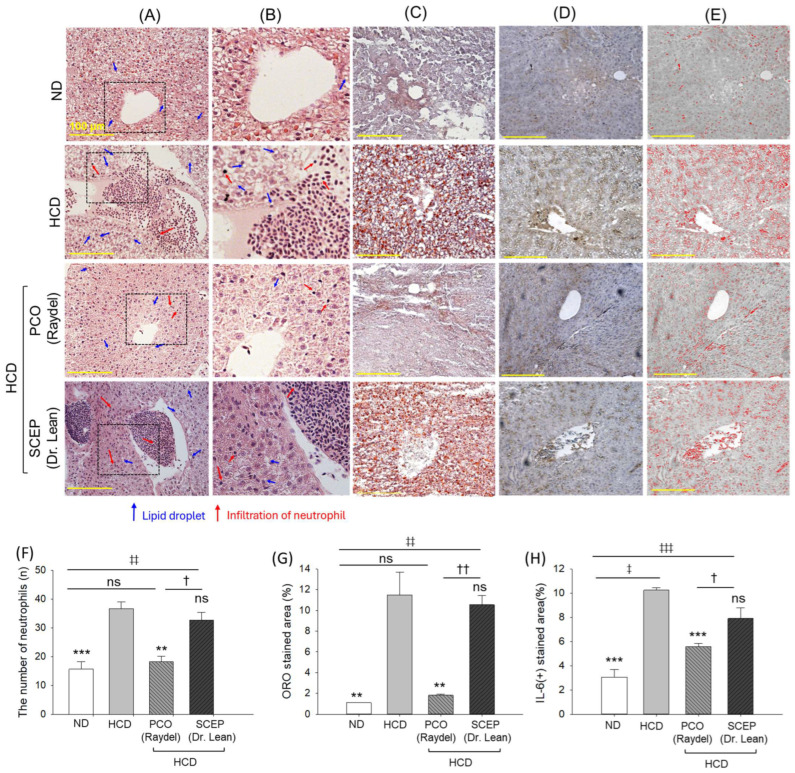
Hepatic histology of zebrafish fed with a high-cholesterol diet (HCD) infused with policosanol (PCO) or sugarcane extract powder (SCEP) for 14 weeks. (**A**) Hematoxylin and eosin (H&E) staining, (**B**) magnified view of H&E section covered with a dotted black box, (**C**) Oil Red O (ORO) staining, (**D**) immunohistochemical staining for the detection of interleukin 6 (IL-6), and (**E**) to enhance the visibility of the IL-6-stained area (brown color) interchanged with red color at the brown threshold value (20–120) using ImageJ software (https://imagej.net/ij, 1.53 version, accessed on 6 June 2023) [scale bar 100 μm]. (**F**) Neutrophil counts, (**G**,**H**) quantification of ORO and IL-6-stained area. Abbreviations: ND: normal tetrabit-fed group; HCD: high-cholesterol diet group; HCD + PCO or SCEP: high-cholesterol diet infused with policosanol (Raydel^®^) or sugarcane extract powder (Dr. Lean) group. The signs ** (*p* < 0.01), *** (*p* < 0.001) indicate statistical difference vs. HCD group; ^‡^ (*p* < 0.05), ^‡‡^ (*p* < 0.01), ^‡‡‡^ (*p* < 0.001) indicate statistical difference vs. ND group, and ^†^ (*p* < 0.05), ^††^ (*p* < 0.01) indicate statistical difference vs. PCO group. “ns” represents a non-significant difference between the groups.

H&E staining of the liver section revealed a 2.4-fold (*p* < 0.001) higher neutrophil count in the HCD-consumed group than in the ND group (Figure 6A,B,F). The supplementation of PCO effectively protected against HCD-triggered hepatic neutrophil infiltration, as evidenced by a 1.9-fold (*p* < 0.01) significantly reduced neutrophil count compared to the HCD group. By contrast, SCEP supplementation was found to be ineffective in inhibiting HCD-induced neutrophil infiltrations.

The highest lipid accumulation, reflected in a maximum ORO-stained area (11.5 ± 2.1%), was detected in the liver of the HCD-consumed group (Figure 6C,G). A reduced ORO-stained area (1.8 ± 0.1%) was observed in the PCO-supplemented group, which was significantly lower by 6.3-fold (*p* < 0.01) than the ORO-stained area observed in the HCD group. SCEP supplementation had a non-significant (*p* > 0.05) effect in inhibiting HCD-induced hepatic lipid accumulation.

Consistent with the findings of the ORO staining, the IHC analysis revealed higher IL-6 production in the HCD group, which was 3.4-fold higher than the IL-6 level in the ND group (Figure 6D,E,H). Supplementation of PCO resulted in a 1.8-fold (*p* < 0.001) reduced IL-6-stained area compared to the HCD group, underscoring the impact of PCO in ameliorating the HCD-provoked IL-6 production. No protective effect of SCEP supplementation was observed against the HCD-induced hepatic IL-6 production. The findings from hepatic histology and IHC staining together confirm that PCO successfully counteracted the negative impacts of HCD. In contrast, SCEP did not exhibit a favorable effect against HCD-related outcomes.

### 3.6. Generation of Reactive Oxygen Species (ROS) and Senescence in the Hepatic Tissue

**Figure 7:**
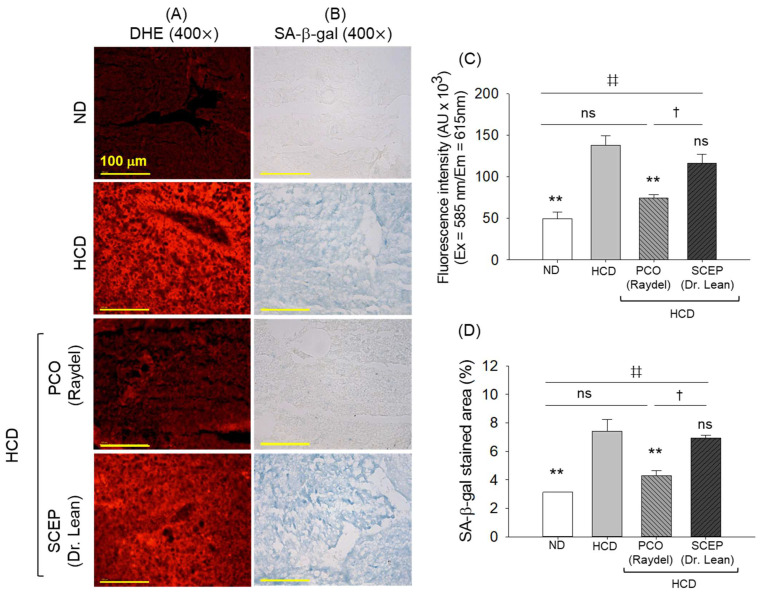
Hepatic (**A**) dihydroethidium (DHE), and (**B**) senescence-associated β-galactosidase (SA-β-gal) staining of zebrafish fed with a high-cholesterol diet (HCD) infused with policosanol (PCO) or sugarcane extract powder (SCEP) for 14 weeks; [scale bar 100 μm] (**C**,**D**) Quantification of the DHE fluorescent intensity and SA-β-gal-stained area. Abbreviations: ND: normal tetrabit-fed group; HCD: high-cholesterol diet group; HCD + PCO or SCEP: high-cholesterol diet infused with policosanol (Raydel^®^) or sugarcane extract powder (Dr. Lean) group. The sign ** (*p* < 0.01) indicates statistical difference vs. HCD group; ^‡‡^ (*p* < 0.01) indicates statistical difference vs. ND group, and ^†^ (*p* < 0.05) indicates statistical difference vs. PCO group. “ns” represents a non-significant difference between the groups.

DHE and SA-β-gal staining revealed a statistically significant higher prevalence of reactive oxygen species (ROS) and senescence-positive cells in the HCD-consumed group, 2.8-fold (*p* < 0.01) and 2.4-fold (*p* < 0.01) higher, respectively, compared to the ND group (Figure 7A–D). The consumption of PCO showed a notable protective effect and attenuated ROS generation and cellular senescence by 1.9-fold (*p* < 0.01) and 1.7-fold (*p* < 0.01), respectively, compared to the HCD group. The quantitative output of DHE and SA-β-gal staining revealed a marginal reduction in ROS and senescence in the SCEP-supplemented group; however, the effects were statistically non-significant (*p* > 0.05) compared to the HCD group.

### 3.7. Kidney Histology

**Figure 8:**
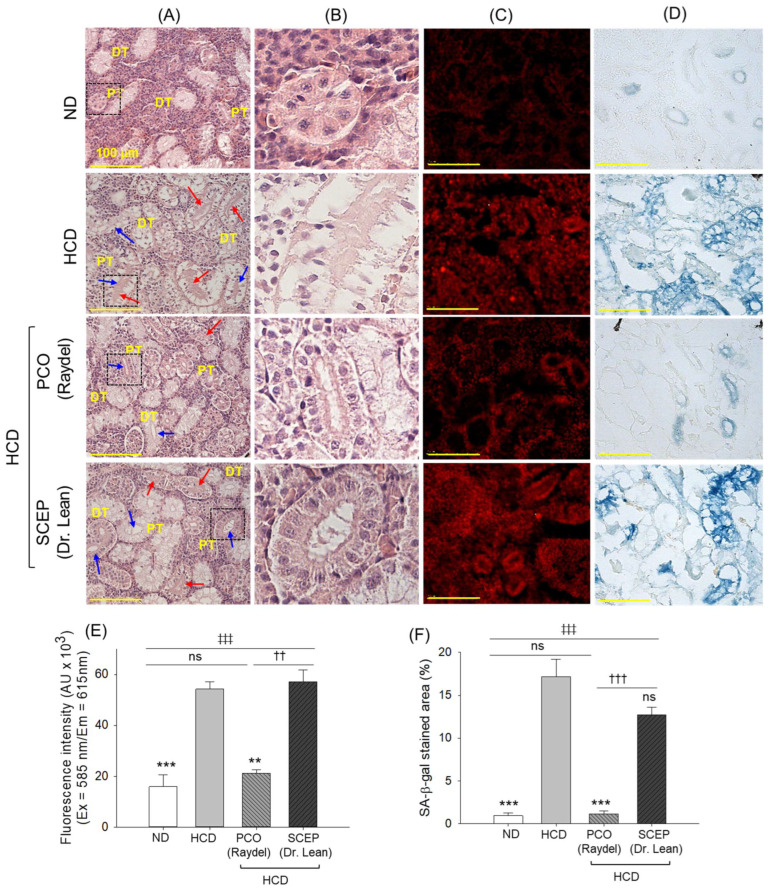
Kidney histology of zebrafish fed with a high-cholesterol diet (HCD) infused with policosanol (PCO) or sugarcane extract powder (SCEP) for 14 weeks. (**A**) Hematoxylin and eosin (H&E) staining, DT and PT representing the distal and proximal tubules. Blue and red arrows highlight the dilated tubular lumen and luminal debris, respectively. (**B**) Magnified view of H&E section covered with a dotted black box. (**C**) Dihydroethidium (DHE), and (**D**) senescence-associated β-galactosidase (SA-β-gal) staining [Scale bar 100 μm]. (**E**,**F**) Quantification of the DHE fluorescent intensity and SA-β-gal-stained area, respectively. Abbreviations: ND: normal tetrabit-fed group; HCD: high-cholesterol diet group; HCD + PCO or SCEP: high-cholesterol diet infused with policosanol (Raydel^®^) or sugarcane extract powder (Dr. Lean) groups. The signs ** (*p* < 0.01), *** (*p* < 0.001) indicate statistical difference vs. HCD group; ^‡‡‡^ (*p* < 0.001) indicate statistical difference vs. ND group, and ^††^ (*p* < 0.01), ^†††^ (*p* < 0.001) indicate statistical difference vs. PCO group. “ns” represents a non-significant difference between the groups.

H&E staining of the kidney tissue section from the HCD group demonstrated structural disruptions in both the distal and proximal tubules, characterized by loss of organized architecture, frequent tubular lumen dilation, and the accumulation of cellular debris within the tubular cast (Figure 8A,B). Supplementation of PCO restricted the histological changes induced by HCD, preserving the overall integrity of the proximal and distal tubules; however, the presence of a dilated tubular lumen and luminal debris was observed in some areas. In contrast, SCEP did not exhibit any substantial effect in protecting kidney impairment caused by the consumption of HCD.

DHE fluorescent staining revealed massive ROS production in the HCD-consumed group that remained unaffected following SCEP supplementation. Notably, supplementation with PCO effectively diminished HCD-elevated ROS production (Figure 8C,E), reflected in a significant 2.5-fold (*p* < 0.01) reduced DHE fluorescent intensity compared to the HCD group. Similar to the outcomes of DHE fluorescent staining, SA-β-gal staining revealed that SCEP was ineffective in preventing HCD-triggered senescence. In contrast, a substantial 14.3-fold (*p* < 0.001) reduction in senescence-positive cells was observed in the PCO-supplemented group compared to the HCD group (Figure 8D,F).

### 3.8. Brain Histology and Immunohistochemistry (IHC)

**Figure 9:**
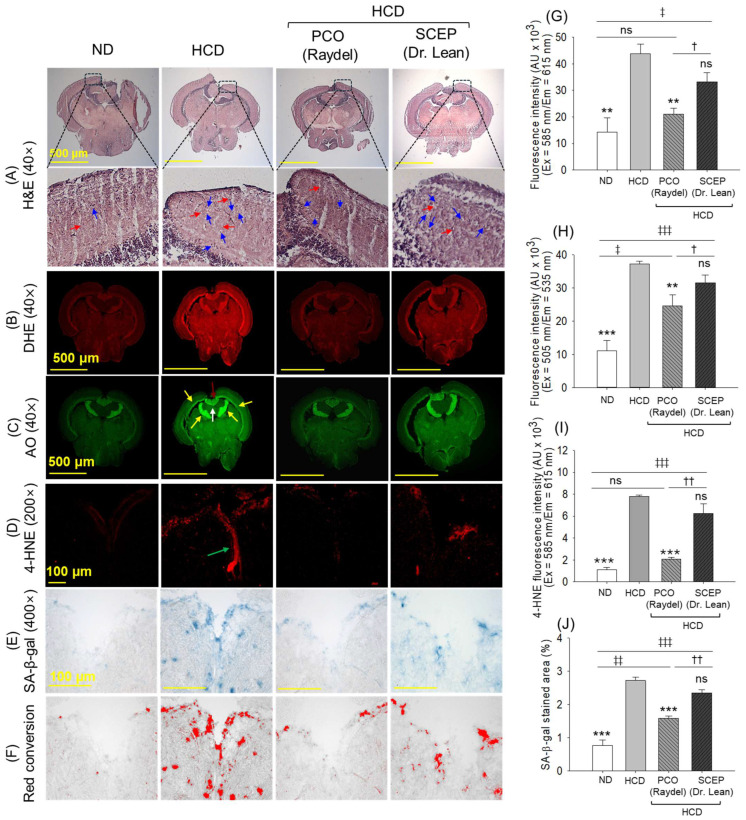
Effect of high-cholesterol diet (HCD) infused with policosanol (PCO) or sugarcane extract powder product (SCEP) on the brain of adult zebrafish during 14 weeks of consumption. (**A**) Hematoxylin and eosin (H&E) staining [40× magnification, scale bar 500 μm]. The red arrows indicate vacuolation, while blue arrows indicate the number of mononuclear cells with a clear zone. (**B**,**C**) Dihydroethidium (DHE) and acridine orange (AO) fluorescent images; the yellow arrow represents the periventricular grey zone (PGZ) of the tectum opticum (TeO), the brown arrow indicates the torus longitudinalis (LT), and the white arrow represents the lateral division of the valvular cerebelli (Val). (**D**) 4-Hydroxynonenal (4-HNE) fluorescent staining; and green arrow, the vascular lacuna of the area postrema (Vas). (**E**) Senescence-associated β-galactosidase (SA-β-gal) staining [scale bar = 100 μm]. (**F**) Red conversion of the SA-β-gal-stained area using the ImageJ software (at blue color threshold value 0–120) to improve the visibility of the SA-β-gal-stained area. (**G**–**I**) Quantification of DHE, AO, and 4-HNE fluorescent intensities, respectively. (**J**) SA-β-gal-stained area. Abbreviations: ND: normal tetrabit-fed group; HCD: high-cholesterol diet group; HCD + PCO or SCEP: high-cholesterol diet infused with policosanol (Raydel^®^) or sugarcane extract powder (Dr. Lean) group. The signs ** (*p* < 0.01), *** (*p* < 0.001) indicate statistical difference vs. HCD group; ^‡^ (*p* < 0.05), ^‡‡^ (*p* < 0.01), ^‡‡‡^ (*p* < 0.001) indicate statistical difference vs. ND group, and ^†^ (*p* < 0.05), ^††^ (*p* < 0.01) indicate statistical difference vs. PCO group. “ns” represents the non-significant difference between the groups.

The H&E staining shown in Figure 9A (and Supplementary Materials Figure S2) revealed vacuolation (highlighted by red arrow) and mononuclear cells with distinct clear zone (highlighted by blue arrow) in the tectum optic (TeO) and periventricular grey zone (PGZ) of the brain section from the HCD group. In comparison, the brain tissue from the PCO-consumed group exhibited an appreciably lower presence of vacuolation and mononuclear cells in these regions. In contrast, SCEP supplementation had a minimal impact on mitigating HCD-induced vacuolation and mononuclear cell accumulation in the TeO and PGZ.

In the brain of the HCD-consumed group, a greater extent of ROS and apoptosis was observed in the PGZ of TeO near the torus longitudinalis (LT) and lateral division of the valvular cerebelli (Val) that represented 3.1-fold (*p* < 0.01) and 3.3-fold (*p* < 0.001) higher levels of ROS and apoptosis than the ND group, respectively (Figure 9B,C,G,H). HCD-augmented ROS and apoptosis diminished by 2.1-fold (*p* < 0.01) and 1.5-fold (*p* < 0.05), respectively, following PCO supplementation.

Consistent with these observations, IHC staining demonstrated a higher accumulation of 4-hydroxynonenal (4-HNE), mainly in the vascular lacuna of the area postrema (Vas) in the brain section of the HCD group, that was~6-fold higher than the 4-HNE level quantified in the brain of the ND group (Figure 9D,I). The HCD-augmented 4-HNE accumulation reduced by 3.6-fold (*p* < 0.05) following PCO supplementation, while a non-significant effect was observed in the SCEP-supplemented group.

As with the IHC staining, a high prevalence of cellular senescence around the Vas was observed in the HCD-supplemented group, which was effectively reduced by 1.6-fold (*p* < 0.05) by PCO supplementation (Figure 9E,F,J). In combination, the results obtained from brain histology highlight the efficacy of PCO in protecting the brain against HCD-induced events, while no protective effect of SCEP was observed.

## 4. Discussion

The effect of PCO purified from Cuban sugarcane wax was compared with whole ground sugarcane extract powder (SCEP) containing RYR in a hyperlipidemic zebrafish model to assess their relative impact on HCD-induced metabolic stress and organ health. After 14-week supplementation, zebrafish in the PCO-supplemented group exhibited survival rates similar to the ND (control) group, underlying the protective role of PCO against HCD-triggered death. In contrast, SCEP showed almost no protective effects against HCD-induced toxicity. While both PCO and SCEP supplementation mitigated HCD-induced body weight gain, PCO demonstrated a significantly greater effect. These findings align with previous studies that reported PCO efficacy in reducing body weight [17,33]. Furthermore, clinical studies on obese participants have also confirmed a substantial role of PCO in weight reduction [34,35]. Mechanistically, PCO enhances adipose tissue energy expenditure [17,36] and stimulates brown adipose tissue activity, contributing to its protective effect against diet-induced obesity [37].

Hyperlipidemia in connection with HCD has been described in various model organisms [38,39,40], and similarly, we observed a disturbed blood lipid profile, marked by elevated TC and TG, and reduced HDL-C levels, in response to HCD. PCO displayed remarkably superior protective effects to SCEP in reducing plasma TC and TG levels and in raising HDL-C levels under HCD following 14 weeks of supplementation. Several studies have suggested the effective role of PCO in preventing TC and TG; however, the efficacy observed varied based on the source material and the composition of the LCAA of PCO. PCO impacts dyslipidemia via a variety of mechanisms, including via a substantial inhibitory effect on 3-hydroxy-3 methyl-glutaryl-coenzyme (HMG-CoA) reductase, a main rate-limiting enzyme in cholesterol biosynthesis [17,36]. The high prevalence of LCAAs, like triacontanol, hexacosanol, and octacosanol, in PCO [23] plays a key role in lowering cholesterol [41,42,43].

Furthermore, PCO positively affects cholesterol efflux capacity [44], and cholesterol catabolism in the liver [21,45] has been determined to prevent excess cholesterol. The cholesterol-lowering effect of SCEP can be attributed to the presence of RYR, which is known to contain monacolin K (identical to lovastatin), and which has been recognized to inhibit HMG-CoA reductase, consequently impacting cholesterol biosynthesis [16]. The HDL-C level was elevated only in response to PCO supplementation, which was remarkably higher than the HDL-C level compared to SCEP. This findings aligns with the previous reports documenting PCO dominance over the RYR product in the augmentation of HDL-C [26]. In another comparative study with type 2 diabetes and hyperlipidemic individuals, PCO displayed higher HDL-C levels than lovastatin [46], supporting the present findings. Among the varied events responsible for HDL-C elevation, the distinctive impact of PCO on cholesteryl ester transfer protein (CETP) inhibition has been documented as a fundamental reason behind HDL-C elevation [35].

Cholesterol and high fat have been associated with elevated blood glucose levels [47], and correspondingly, we observed an elevated blood glucose level in the HCD-consumed group that was substantially mitigated by supplementation of PCO, while SCEP showed a non-significant effect. The results obtained from PCO supplementation on the glucose level are consistent with earlier findings documenting the effect of PCO in controlling blood glucose levels in zebrafish and humans [18], mediated by the PCO effect on insulin sensitization and secretion [18]. MDA is an important oxidative stress marker [48]; likewise, sulfhydryl content has been recognized as an oxidative stress marker, and diminished levels have been associated with various diseases [49,50,51]. HCD provoked higher MDA levels, and low sulfhydryl content was substantially restored by consumption of PCO, confirming its protective effect in reducing HCD-induced oxidative stress. Correspondingly, restoration of HCD-diminished antioxidant status assessed by FRA and PON activity was observed in response to PCO; these findings are in good agreement with the literature documenting the positive effect of PCO on MDA [20,35], FRA, and PON activity [26].

A high-cholesterol diet and dyslipidemia also adversely affect the liver [52], and we also observed noticeable liver damage, enhanced neutrophil infiltration, fatty liver changes, and higher inflammation (IL-6 production) in response to HCD consumption. HCD-induced changes were substantially reverted by the consumption of PCO, consistent with earlier reports indicating the hepatoprotective nature of policosanol against external stress [20,53]. Notably, hexacosanol (an important LCAA in PCO) has been documented to protect against lipid accumulation by inhibiting autophagy [41], which has been recognized as a key event for the inhibition of lipid accumulation and fatty liver changes (steatosis) [54]. In contrast to PCO, no visible effect of SCEP was observed with respect to hepatoprotection, probably owing to the presence of RYR, which has been recognized to damage the liver [55,56].

DHE and AO staining of the hepatic section demonstrated the effectiveness of PCO in mitigating HCD-induced ROS generation and apoptosis. This protective effect is attributed to the inherent cellular antioxidant properties of PCO [22,24]. Specifically, triacontanol (an LCAA in PCO) has been reported to prevent lipid peroxidation and oxidative stress through its antioxidant activity [17], further supporting the current findings. Like its role in suppressing ROS generation, PCO consumption diminished apoptosis and senescence, which can be attributed to lower ROS levels in the presence of PCO. ROS-mediated oxidative stress is a critical factor in provoking cellular senescence [57,58]; this effect underscores the protective mechanism of PCO against HCD-induced senescence.

High cholesterol consumption has been documented to damage the kidneys [59], and we observed the kidney-damaging effect of HCD. Consistent with its protective effects on the liver, PCO supplementation mitigated HCD-induced kidney impairment by reducing ROS generation and senescence. In contrast, SCEP did not exhibit any protective effects, potentially due to the presence of RYR, which has been implicated in kidney impairment [60]. The substantial kidney protective effects of PCO are likely attributable to its ability to inhibit oxidative stress, a key contributor to kidney damage [61].

In line with its protective role in the liver and kidneys, PCO demonstrated a substantial protective effect on the brain against HCD-provoked events. PCO supplementation reduced cellular senescence, ROS levels, and apoptosis in the brain, whereas SCEP supplementation showed no such effect. In accordance with DHE staining results, IHC staining revealed a pronounced accumulation of the lipid peroxidation aldehyde product 4-HNE [62] in the HCD group, underscoring heightened oxidative stress in the brain. Notably, PCO supplementation effectively protected HCD-triggered 4-HNE generation and accumulation in the brain, while no effect of SCEP supplementation was observed in mitigating 4-HNE generation. By reducing ROS generation, PCO also mitigated apoptosis and senescence, as oxidative stress is directly associated with apoptosis [63,64] and senescence [57,58]. Furthermore, PCO has been linked to the inhibition of caspase-3, an important enzyme that initiates the apoptotic process [65]. In contrast, SCEP showed no inhibitory effect on ROS, apoptosis, or senescence, likely due to the presence of RYR (that contains monacolin K), which has been documented to induce oxidative stress-mediated apoptosis via regulating mitogen-activated protein kinase and nuclear factor-κB signaling [66]. Also, the brain-protective effect of PCO is closely linked to its protective role in the liver and kidneys, reinforcing the concept of the liver–brain [67] and kidney–brain axis [68,69]. Previous reports suggest that excessive lipid accumulation in the liver alters the composition and amounts of circulating fat-derived products, compromising the blood–brain barrier and facilitating the accumulation of toxic metabolites and inflammatory cells in the brain [70], leading to brain damage. Likewise, oxidative stress and chronic kidney disease contribute to cognitive impairment and neuropsychiatric disorders [68], highlighting the kidney–brain connection. By safeguarding liver and kidney function, PCO indirectly protects the brain, mitigating damage through these interrelated pathways.

## 5. Conclusions

In a 14-week comparative study, policosanol (PCO, Raydel^®^) showed significantly higher efficacy than sugarcane extract powder (SCEP, Dr. Lean) in mitigating HCD-triggered changes in the plasma lipid profile and antioxidant parameters. Furthermore, PCO effectively mitigated HCD-augmented blood glucose, oxidative stress, and hepatic function biomarkers, while SCEP failed to produce such events. PCO also attenuated hepatic steatosis and IL-6 expression and protected the kidneys against oxidative damage and cellular senescence. It also prevented reactive 4-HNE accumulation in the brain, safeguarding the brain from oxidative injury and apoptosis. The findings uncover the functional discrepancy between PCO and SCEP, establishing PCO’s functional superiority against HCD-induced metabolic and oxidative disturbances.

## Data Availability

The data used to support the findings of this study are available from the corresponding author upon reasonable request.

## Supplementary Materials

The supporting information can be downloaded at: https://www.mdpi.com/article/10.3390/cimb47050354/s1.
