# Supplementary material for: Effect of 14-Week Supplementation of Highly Purified Policosanol (Raydel®) and a Sugar Cane Extract Powder (SCEP) on Dyslipidemia and Oxidative Variables in Hyperlipidemic Zebrafish: Insight into Liver, Kidney, and Brain Health
Source: Curr Issues Mol Biol. 2025 May 13;47(5):354. doi: 10.3390/cimb47050354 (PMC12109632; doi:10.3390/cimb47050354)
Supplement: Supplementary file 1 [file cimb-47-00354-s001.zip › cimb-3626728-supplementary.pdf]

Supplementary Material

1. List of the used chemicals: Dihydroethidium (DHE, 104821-25-2, Cat #37291), and acridine orange (AO, 65-61-2, Cat#A9231), oil red O (Cat#O0625), and 2-phenoxyethanol (Sigma P1126; St. Louis, MO, USA), paraoxon-ethyl (Cat. No. 36186) and 5-bromo-4-chloro-3-indolyl β D-galactopyranoside (X-gal, Cat#B54252) were procured from Sigma–Aldrich (St. Louis, MO, USA). All other chemicals and reagents else otherwise stated were of analytical grade and used as supplied.

2. Quantification of blood lipoprotein profile hepatic function biomarkers AST and ALT

The plasma total cholesterol (TC) and triglycerides (TGs) were determined using commercial assay kits (cholesterol, T-CHO, and TGs, Cleantech TS-S; Wako Pure Chemical, Osaka, Japan) as per the method suggested by the suppliers. In brief, 5 μL serum was mixed with 200 μL reaction mixture (supplied with a commercial assay kit) for the TC analysis. The content was incubated at 37°C for 10 min, resulting in a red-colored product quantified by adsorption at 490 nm (Microplate reader, Bio-Rad, Hercules, CA, USA).

Similarly, 5 μL serum was mixed with a 200 μL of TGs-specific reaction mixture (supplied with a commercial assay kit) for TGs analysis. The content was incubated for 10 min at 37°C, and the formed colored product was quantified by taking adsorption at 490 nm.

For HDL-C analysis, serum was mixed in an equal ratio with the separation solution (supplied with a commercial assay kit), followed by centrifugation at 3,000 rpm for 10 min. The supernatant (20 μL) was collected and blended with a 200 μL reaction mixture (supplied with a commercial assay kit). After 10 min incubation at 37°C, red color intensity corresponding to HDL-C was quantified by taking absorption at 490 nm.

The commercial diagnostic kit (Asan Pharmaceutical, Hwasung, Republic of Korea) was used to quantify aspartate transaminase (AST) and alanine transaminase (ALT) levels in the plasma, following the instructions suggested by the manufacturers. Briefly, 5 μL of plasma was combined with 250 μL of either AST or ALT-specific solution, as supplied in the diagnostic kit. Following a 30 min incubation for AST or 60 min incubation of ALT at 37°C, the mixture was then blended with 250 μL of the respective coloring reagent (AST or ATL-specific, provided in the diagnostic kit). After a subsequent 20 min incubation at room temperature, 250 μL of 0.4 N NaOH was introduced to halt the reaction. Finally, the AST and ATL were quantified by measuring absorbance at 490 nm.

Supplementary Table S1.

RAYDEL
Health Confidence

Raydel Australia Pty Ltd
Level 1, building 1, 9-15 Chilvers Rd.
Thornleigh NSW 2120 Australia
Tel +61 2 9480 1300
Fax +61 2 9480 1399
A.B.N. 45 054 555 903
www.raydel.com.au
info@raydel.com.au

Product name: Policosanol

Batch #: 310030324

Date of Manufacture: 12/03/2024

Parameter	Results	Approved Limits
Color	Complies	Off white to cream
**Identity and Purity***		
1-tetracosanol (C_{24})	0.05 %	0.00 – 2.0 %
1-hexacosanol (C_{26})	3.34 %	3.0 –10.0 %
1-heptacosanol (C_{27})	0.82 %	0.1 - 3.0 %
1-octacosanol (C_{28})	60.02 %	60.0 –70.0 %
1-nonacosanol (C_{29})	0.55 %	0.1- 2.0 %
1-triacontanol (C_{30})	14.96 %	10.0 – 15.0%
1-dotriacontanol (C_{32})	8.24 %	5.0 - 10.0 %
1-tetratriacontanol (C_{34})	2.39 %	0.1 – 5.0 %
**Total (Purity*)**	**90.37 %**	**≥ 90 %**
**Other quality specifications**		
**Melting temperature**	81.3-83.0 °C	78.0 – 83.0 °C
**Loss on drying**	0.54 %	≤ 1.0 %
**Residue of ignition**	0.72 %	≤ 0.85 %
**Heavy metals (Pb, Cd, Hg)**	<0.000015 %	≤ 0.001 %
**Sodium content**	86.34 ppm	≤ 100 ppm
**Potassium content**	2905.41 ppm	≤ 4500 ppm
**Residual solvents**		
**Acetone**	≤ 0.03	≤ 0.03 g/kg
**Hexane**	≤ 0.005	≤ 0.005 g/kg
**Microbiological content**		
**Total Aerobic Microbial Count**	≤10	≤10^3 per g
**Yeast and mould**	≤10	≤10^2 per g
**Enterobacteria or Coliform count**	≤10	≤10^2 per g
***Staphylococcus aureus, Pseudomonas aeruginosa, Escherichia* coli, Candida albicans**	Absent	Absent in 1 g
Salmonella sp	Absent	Absent in 10 g
Observations:		
**References:**	* Manufacturer GC validated method, purity expressed as the total of high molecular weight alcohols	

Note about storage conditions: No special storage conditions are required. The substance has a shelf life of 5 years stored under ambient conditions of climatic Zones IV or II, as demonstrated in stability studies performed according to ICH guidelines.

Approved (_X_) Released (_X_) Rejected (___)

This COA is it reproduced from supplier's COA

Table S1: Certificate of analysis and the composition of the policosanol used.

Supplementary Table S2.

S.N	Name of the major ingredients	Amount
1.	Sugarcane wax alcohol extract powder	Not disclosed
2.	Red yeast rice powder	Not disclosed
3.	Prebiotics mixed powder	Not disclosed
4.	Cotton seed oil	Not disclosed
5.	Corn protein extract	Not disclosed
6.	Green tea powder	Not disclosed
7.	Lemon extract powder	Not disclosed

Table S2: Details of the major ingredients of sugar cane extracted powder (SCEP, Dr. Lean).

Supplementary figure S1.

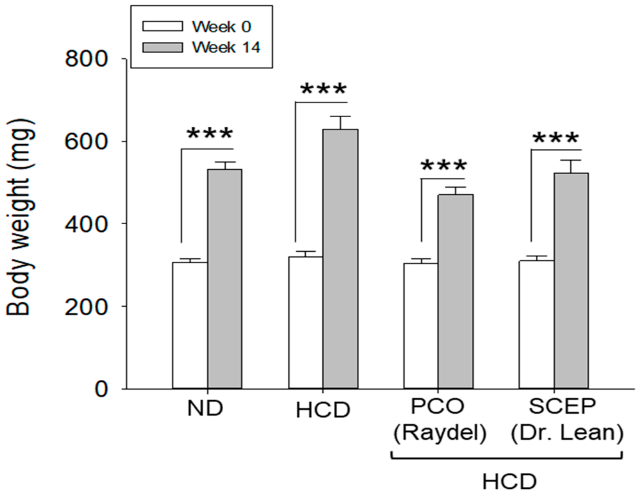

Supplementary Figure S1. Effect of high cholesterol diet (HCD) infused with policosanol (PCO) or sugarcane extract powder (SCEP) on the body weight of the adult zebrafish at week 0- and 14-weeks. The repeated measure ANOVA was utilized to establish the statistical difference in the body weight with respect to time, different diets and their interaction. The sign *** (p<0.001) highlights the statistical difference among the groups between the body weigh changes week 0 and week 14. Abbreviation ND: normal tetrabit fed group, HCD: high cholesterol diet group, HCD+PCO or SCEP: high cholesterol diet infused with policosanol (Raydel) or sugarcane extract powder (Dr. Lean) group.

Supplementary figure S2.

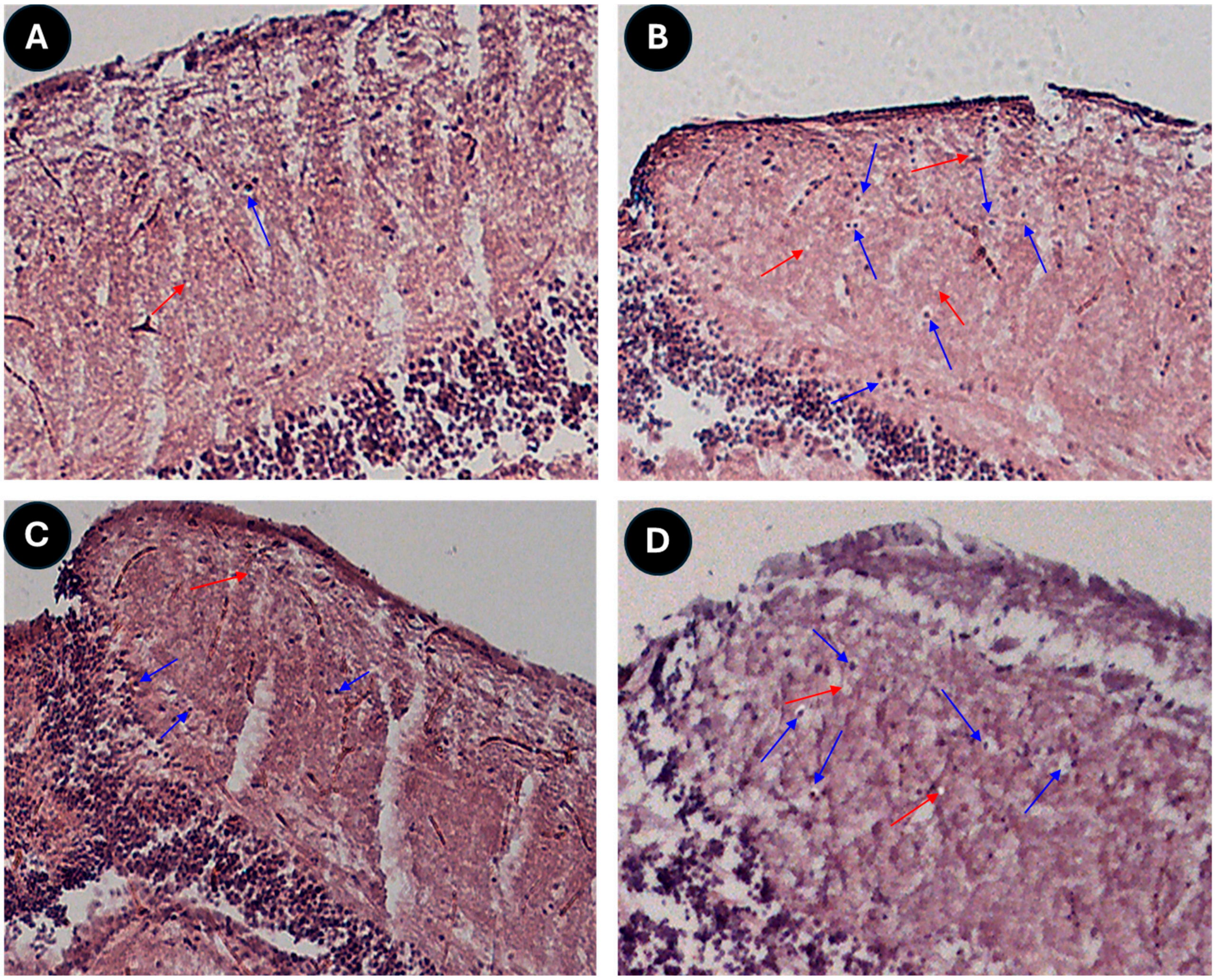

Supplementary Figure S2. A magnified view of hematoxylin and eosin (H&E) stained area of zebrafish brain section following 14 weeks supplementation of (A) ND, (B) HCD, (C) HCD+PCO and (D) HCD+SCEP. The red and blue arrow highlights vacuolation and mononuclear cells with distinct, clear zones, respectively. Abbreviation ND: normal tetrabit fed group, HCD: high cholesterol diet group, HCD+PCO or SCEP: high cholesterol diet infused with policosanol (Raydel) or sugarcane extract powder (Dr. Lean) group.
